# Case Report: Fatal pneumonia as a manifestation of X-linked hypohidrotic ectodermal dysplasia: clinical phenotype and genotype characterization of a novel *EDA* frameshift variant in a four-generation pedigree

**DOI:** 10.3389/fped.2026.1799743

**Published:** 2026-06-03

**Authors:** Huan Liu, Ruiping Zhao, Huili Liu, Wenxuan Duan, Yanhu Li, Bin Jin, Yigu Gong

**Affiliations:** 1 The First Hospital, Lanzhou University, Lanzhou, Gansu, China; 2 The Second Hospital, Lanzhou University, Lanzhou, Gansu, China

**Keywords:** bronchial gland hypoplasia, EDA, frameshift variant, lethal outcome, pneumonia, X-linked hypohidrotic ectodermal dysplasia

## Abstract

**Background:**

Clinically, X-linked hypohidrotic ectodermal dysplasia is identified by its classic triad of hypohidrosis, hypotrichosis, and oligodontia. Nevertheless, the true threat to patient survival often stems from respiratory complications that extend beyond these obvious ectodermal traits. Mechanistically, ectodysplasin A *(EDA)* variants impair skin appendages and respiratory submucosal glands, undermining mucociliary clearance. This vulnerability, exacerbated by thermoregulatory instability from sweat gland dysfunction, establishes a pathological environment prone to severe infections, leaving infants at high risk for life-threatening fungal pneumonia and sepsis. Despite high early-life mortality, this potential lethality is frequently overlooked in pediatrics because glandular defects are nearly invisible on standard imaging.

**Case presentation:**

The proband was a male infant aged approximately 74 days. After birth, he repeatedly developed a fever and was hospitalized. Imaging confirmed pulmonary infection, and pathogen examination revealed *Fusarium*, *Saccharomyces cerevisiae*, and *Achromobacter xylosoxidans*. Given the patient's typical clinical features of ectodermal dysplasia, whole exome sequencing was performed with consent. Results showed a novel hemizygous frameshift variant, c.916delC (p.Gln306ArgfsTer2), in *EDA*. Subsequently, Sanger sequencing was performed on the direct relatives of the proband, and detailed clinical phenotype assessment and medical history collection were conducted for 17 members spanning four generations of the pedigree. Lineage analysis showed that transmission of this variant exhibited perfect cosegregation with the disease phenotype in the family, consistent with X-linked recessive inheritance. Unfortunately, the proband died at 2 years and 6 months from severe pneumonia.

**Conclusion:**

This study identified a novel pathogenic EDA variant c.916delC, expanding the pathogenic variation spectrum of this gene. Through detailed family case reports and combined with imaging and pathogen evidence, we observe and suggest that underdeveloped respiratory mucosal glands and skin heat dissipation disorders may be an important pathological basis for fatal pneumonia and multiple infections in infants and young children with X-linked hypohidrotic ectodermal dysplasia. This discovery emphasizes the necessity of increasing clinical awareness of respiratory system involvement in such children, and suggests that adopting active temperature management, airway care, and reasonable anti-infection strategies in the early stages of diagnosis and treatment has important clinical reference value for improving the prognosis of children.

## 1 Introduction

X-linked hypohidrotic ectodermal dysplasia (XLHED) is a rare X-linked recessive disease with an incidence rate of 1/100000. Although clinical diagnosis mainly relies on the typical triad of “little or no sweat, lack of hair, and missing teeth”, these external features often mask the risk of mortality from the disease. Research has shown that the mortality rate of XLHED in infancy is as high as 30%, and its fatal threat not only comes from the loss of ectodermal development, but also from deep systemic involvement (1). On the one hand, severe pneumonia caused by underdeveloped respiratory glands, as well as heat regulation loss and dehydration caused by sweat gland dysfunction, constitute the main causes of death. On the other hand, physical barrier defects caused by dryness and skin cracking create channels for pathogen invasion, further increasing susceptibility to systemic infections. Unfortunately, these key high-risk factors that truly determine patient prognosis are often overlooked in clinical practice, leading to serious consequences.

This study reports a novel frameshift variant in the *EDA* identified in a pediatric patient from Gansu Province, China, thereby expanding the known pathogenic variant spectrum. By presenting a case of XLHED characterized primarily by fatal pneumonia—supported by comprehensive clinical, radiographic, and microbiological evidence along with a four-generation pedigree analysis—this report underscores that the impact of *EDA* variants extends beyond ectodermal appendages like teeth and hair. It calls attention to the clinical significance of respiratory submucosal gland hypoplasia and the consequent predisposition to severe, potentially life-threatening infections. For individuals with suggestive clinical features or a high-risk family history, early genetic diagnosis may facilitate timely intervention and potentially mitigate the risk of adverse outcomes associated with diagnostic delays.

## 2 Case presentation

The studies involving human participants were approved by the ethics committee of Gansu Provincial Lanzhou University First Hospital (2025-2127). Informed consent was obtained.

**Figure 1:**
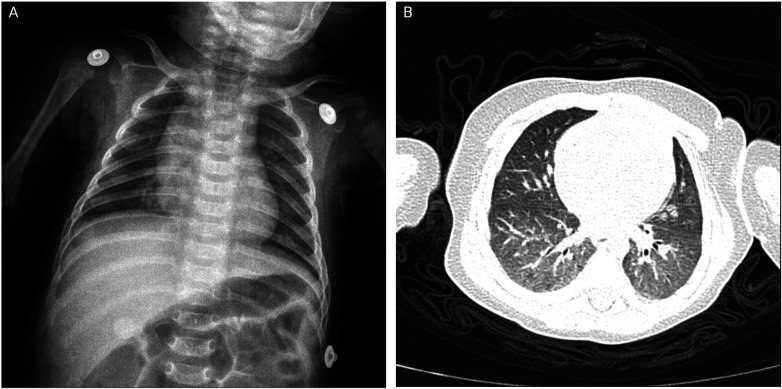
Radiological findings of the patient's respiratory infection. **(A)** Anteroposterior chest radiograph revealing diffuse, patchy infiltrates and increased lung markings bilaterally, suggestive of extensive bronchopneumonia. **(B)** Axial high-resolution computed tomography scan of the chest (lung window) demonstrating multifocal ground-glass opacities, patchy consolidations, and peribronchial thickening, consistent with a severe, multi-lobar pulmonary infection.

**Figure 2:**
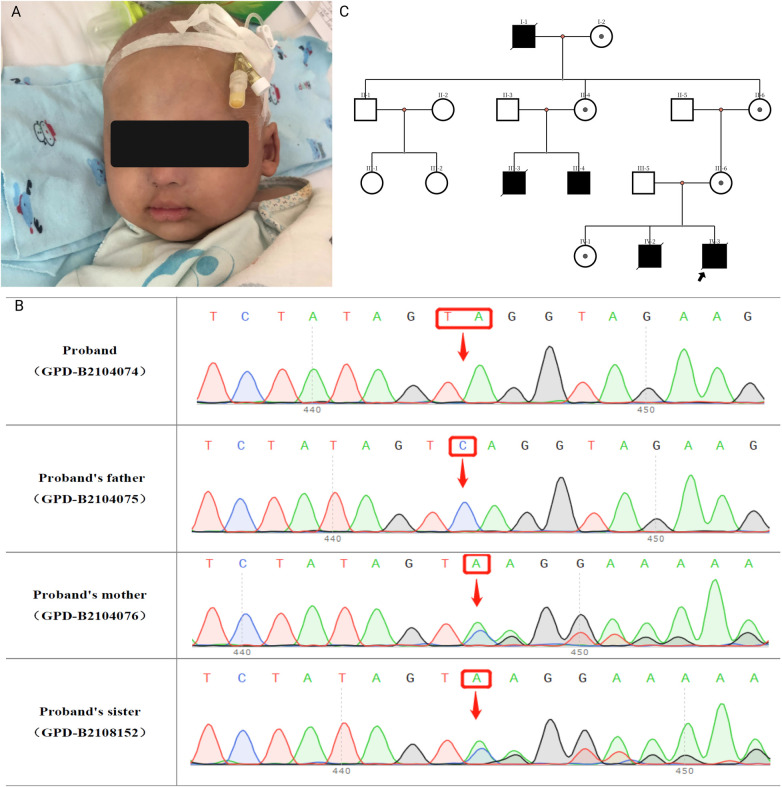
Clinical and genetic findings in the family with XLHED. **(A)** Clinical appearance of the proband, showing sparse hair. **(B)** Sanger sequencing chromatogram of the *EDA*. The arrow indicates the site of the hemizygous c.916delC variant in the proband, compared with the heterozygous carrier (mother) and a wild-type control. **(C)** Pedigree of the family.

The proband was a full-term male infant with a normal birth history. About 20 days after birth, the parents noticed that the child had a fever with a body temperature of 37.8 ℃ and a slight cough. The child was given oral cold medicine. At 74 days of age, he was admitted to our hospital due to recurrent fever for 54 days, worsening cough for 5 days, and mild respiratory distress. Chest X-rays and computed tomography scans showed extensive pulmonary infection, characterized by bilateral patchy infiltration, bronchial wall thickening, and focal consolidation, which, combined with clinical manifestations, confirmed severe pneumonia (Figures 1A,B). In laboratory tests, *Fusarium* and *Saccharomyces cerevisiae* were detected in bronchoalveolar lavage fluid and blood culture, confirming sepsis and fungal pneumonia. In addition, the second-generation metagenomic sequencing of the alveolar lavage fluid metagenome also detected *Achromobacter xylosoxidans*, which accounted for 89.3% of all bacterial sequences and was the absolute dominant bacterium in the sample. After active combined antimicrobial treatment, the proband's lung infection and sepsis improved. However, the proband was unable to dissipate heat through sweating. His body temperature fluctuated between 36.2 °C and 36.9 °C in the morning, and rose to 37.0 °C–38.0 °C at midday and in the afternoon. Relevant examinations excluded other infectious and non-infectious diseases, such as leukemia and connective tissue diseases. Physical examination revealed that the child had rough skin, no sweat during fever, no hair throughout the body, a raised forehead, a small nose, and protruding lips (Figure 2A). Based on these clinical manifestations, hypohidrotic ectodermal dysplasia was highly suspected clinically, and Whole Exome Sequencing (WES) testing was completed.

WES was performed on the proband to clarify the genetic cause. A novel heterozygous variant c.916delC was discovered in exon 7 of the proband *EDA* (NM_001399.5). The single base deletion causes a frameshift and predicts the introduction of an early termination codon (p.Gln306ArgfsTer2) at the second position of the new reading frame. The key molecular mechanism prediction shows that due to the premature termination codon being located within 50 bp of the second to last exon end, according to the ‘50 bp rule’, the mutated mRNA is predicted to escape nonsense mediated degradation, resulting in a truncated protein that retains some structure but loses the C-terminal key functional domain (tumor necrosis factor like domain), leading to the loss of its biological function (2). Sanger sequencing confirmed the variant in the proband and determined that their mother and sister were heterozygous carriers, while their father was wild-type (Figure 2B). This hemizygous variant, located at chrX:69,253,370 (GRCh37/hg19), was maternally inherited.

To further verify the pathogenicity and genetic pattern of this variant in the family, we conducted detailed phenotypic characterization and lineage mapping of 17 members across four generations (Figure 2C). Clinical investigations have shown that this variant exhibits high phenotypic consistency and gender differences throughout the entire family. Five male affected individuals in the family all exhibited typical XLHED characteristics, accompanied by a high mortality rate of pneumonia. For example, patients I-1, III-3, IV-2, and the proband (IV-3) died of unexplained fever and pneumonia at the ages of 50 years, 3 years, 1 month, and 2 years and 6 months, respectively. The only existing male patient (III-4) is currently 28 years old, with rough skin, no sweat, an inability to tolerate high temperatures, frequent respiratory infections, missing teeth, cone-shaped teeth, and long-term dental treatment for dental caries. II4 and II6 female carriers, without special facial features, normal hair, but with thicker skin, less sweating than normal people, afraid of heat, missing teeth, and loose and falling teeth in their forties. III6 female carriers have normal sweating, delayed sprouting compared to normal children, conical teeth, 2 missing teeth, long-term dental treatment for dental caries, slightly rough skin, and long-term treatment for eczema. At the age of 8, a female carrier of type IV has normal sweating, malocclusion of teeth, and eczema. This molecular-level nuclear family validation corroborates the clinical co-segregation phenomenon of severe male and mild female diseases within the family, jointly confirming that the variant follows the classic X-linked recessive genetic pattern. According to the guidelines of the American Society for Medical Genetics and Genomics, this variant is classified as “pathogenic” (PM2 Supporting, PVS1_Strong, PP4, PP1) (detailed criteria are shown in the supplementary material). This variant has not been previously reported in ClinVar, OMIM, or HGMD databases.

During hospitalization, the medical team implemented active, comprehensive treatment for the patient's recurrent fever and severe pneumonia, including a combination of broad-spectrum antibiotics and antifungal agents, bronchoalveolar lavage, and intravenous immunoglobulin support. After treatment, the child recovered from the pulmonary infection and was discharged smoothly. After discharge, the doctor provided the family with a detailed preventive care plan, including rigorous respiratory care, wearing masks in crowded places, balanced nutrition, and emphasized the strict control of environmental temperature through air conditioning to cope with functional disorders. However, during the nationwide outbreak of Mycoplasma pneumoniae (approximately late 2023, when the proband was 2.5 years old), the child developed a sudden high fever and cough. Due to the limited accessibility of tertiary medical resources at that time and the family's proximity, the child was initially treated at a local primary care clinic. Despite receiving intravenous infusions for two days, his condition deteriorated rapidly without timely pathogen identification or intensive monitoring. By the time he was emergently transferred to the local county hospital, his condition had become irreversible, and he passed away shortly after arrival. The clinical progression and major diagnostic milestones of the proband are summarized in Table 1.

**Table 1:** Patient treatment and follow-up form.

Date	Age	Major clinical events	Healthcare setting	Outcome
2021-03-07	1 d	Full-term birth, no significant abnormalities noted.	Local County Hospital	–
2021-03-27	20 d	Fever and mild cough, treated with oral medication.	Local County Hospital	Worsened
2021-05-20	74 d	Recurrent fever (54 days), progressive cough, and mild dyspnea. Fever persisted after recovery from pneumonia and sepsis. Pedigree-based (4 members) whole-exome sequencing was performed.	First Hospital of Lanzhou University (Provincial Tertiary Hospital)	Discharged upon recovery
2021-06-10	95 d	WES results confirmed diagnosis of XLHED; the family was counseled on the etiology of fever, respiratory hygiene, and thermoregulatory care.	–	–
2022-04-05	1 y, 1 m	Fever and cough for 5 days with mild tachypnea, diagnosed with bronchopneumonia and treated with antibiotics.	Local County Hospital	Resolved
2022-11-13	1 y, 8 m	Eruption of only 2 deciduous teeth (significant oligodontia compared to peers). Language and motor development were consistent with age-appropriate milestones.	–	–
2023-04-08	2 y, 1 m	Recurrent fever and cough for 5 days with mild tachypnea; diagnosed with bronchopneumonia and treated with antibiotics.	Local County Hospital	Resolved
2023-09-05	2 y, 6 m	Fever and cough for 3 days, received outpatient intravenous infusion and oral medication.	Local Medical Clinic	Deceased

d, days; m, months; y, years.

## 3 Discussion

This study identified a c.916delC (p.Gln306ArgfsTer2) hemizygous frameshift variant in exon 7 of the *EDA* in the proband. Through clinical phenotype collection and lineage analysis of 17 members of the fourth-generation family, it was confirmed that this variant was perfectly co-segregated with the XLHED phenotype, consistent with the X-linked recessive inheritance pattern. Through bioinformatics analysis and screening, we excluded potential confounding factors, including primary ciliary dyskinesia and primary immunodeficiency. Unlike previous reports that focused on the triad of sweating, hair loss, and missing teeth, this family reveals a clinical fact with warning significance. There were 5 patients in the family, with a high mortality rate. The age of death is mainly in childhood, and the causes of death for all 4 patients were high fever and pulmonary infection. The first admission of the proband showed severe pneumonia, extensive lung lesions on imaging, and a clinical process of repeated infections until death, all of which are direct manifestations of respiratory defense dysfunction. This phenomenon prompts us to delve deeper into the underlying mechanisms of fatal respiratory lesions in children with XLHED.

We believe that the submucosal glands of the respiratory tract are key target organs for EDA action. The gland loss caused by its variant fundamentally deprives the mucous environment necessary for the operation of the mucosal ciliary clearance system. In the absence of mucus coverage, the respiratory tract loses its ability to physically capture and chemically kill pathogens. The ciliary transport function system is further affected. This environment makes it easy for opportunistic pathogens to colonize the respiratory tract. The pathogen infections identified in this case, such as *Fusarium* and *Saccharomyces* cerevisiae, truly reflect the child's extreme immune susceptibility due to congenital immune defects. This not only greatly increases the complexity of clinical diagnosis and treatment, but also is the core factor leading to the rapid progression of lung lesions and ultimately death. In addition, the multi-organ involvement feature of XLHED further exacerbates the severity of the condition. The impaired skin barrier function caused by developmental disorders of sweat glands and hair follicles significantly increases the risk of systemic infections. The temperature regulation disorder caused by low sweating can trigger uncontrollable high fever during infection, forming a vicious cycle.

In the exploration of genetic patterns, we noticed that some female carriers exhibited mild signs of heterogeneity, such as abnormal tooth development, low sweating, and eczema. These hidden ectodermal signs should not be ignored, as they can serve as important auxiliary evidence for early identification of high-risk families, guiding genetic counseling and screening, and are of great significance for blocking the transmission of such highly lethal variants through prenatal diagnosis.

These clinical inferences have been supported by molecular mechanisms and animal experiments. The EDA/Ectodysplasin A receptor (EDAR)/nuclear factor kappa-B signaling pathway is the core regulator*y* axis for the development of ectodermal appendages, and also participates in the branching morphology of respiratory submucosal glands through epithelial-mesenchymal interactions (3). Del Pozo et al. provided direct evidence that the upper respiratory tract nasal, pharyngeal, and Eustachian tube submucosal glands were completely absent in *EDA*-deficient Tabby mice and *EDAR* gene-deficient downless mice. This structural defect directly led to severe impairment of mucosal ciliary clearance function, resulting in phenotypes such as rhinitis, otitis media, and nasopharyngitis in mice (4). More importantly, in the XLHED dog model, the absence of submucosal glands in the airway has also been confirmed to be the pathological basis for recurrent respiratory infections. Recombinant EDA protein replacement therapy can not only restore missing respiratory glands but also significantly improve mucociliary clearance function and control respiratory infections. Therefore, both animal models and canine studies have shown that the interference of EDA signaling pathway defects on the development of respiratory submucosal glands is the pathophysiological basis for the repeated occurrence of severe pneumonia in children with XLHED.

Pathogenic variants affecting other components of the EDA-EDAR-EDARADD signaling pathway have also been associated with hypohidrotic ectodermal dysplasia and variable clinical severity. For example, a recent report describing a novel homozygous frameshift insertion in EDARADD demonstrated classical HED features in two siblings, further supporting the critical role of nuclear factor kappa-B pathway disruption in ectodermal development (5). Although severe respiratory complications were not the primary focus in that study, the shared molecular pathway highlights how defects in different nodes of the EDA signaling cascade may result in overlapping phenotypes and potentially variable systemic vulnerability. These findings collectively reinforce the importance of comprehensive pathway-based genetic evaluation in patients presenting with HED features.

The second-generation metagenomic sequencing results of this case provide direct clinical evidence for this mechanism. The dominant bacterium in the bronchoalveolar lavage fluid specimen of the proband is *Achromobacter xylosoxidans*, which accounts for 89.3% of all bacterial sequences. This bacterium is a clear opportunistic pathogen that can cause severe pneumonia and bacteremia in immunocompromised patients and those with underlying lung diseases such as cystic fibrosis and bronchiectasis (6, 7). Meanwhile, its natural resistance to multiple antibiotics makes post-infection treatment extremely difficult (8). The dominant position of this bacterium in the bronchoalveolar lavage fluid of this case suggests that the ciliary clearance function of the upper respiratory mucosa in the child has been severely impaired, which is highly consistent with XLHED-specific respiratory gland dysplasia. Therefore, the fatal pneumonia of the proband is not simply a case of “aspiration after fever”, but more likely a secondary infection caused by congenital defects in the respiratory mucosal barrier and clearance function, which can be mutually confirmed with classical theoretical models at the clinical level.

XLHED is a multi-system involvement disease that requires multidisciplinary whole life cycle management (MDT management model). The focus of intervention varies at different age stages. For example, in adulthood, the key focus is on dental issues, while in childhood, the focus is on infection and temperature management to prevent high fever and fatal pneumonia. Given the high risk of bacterial and fungal infections and the associated mortality in patients with XLHED, it may be necessary to adopt more proactive and comprehensive anti-infection strategies in clinical management, including early use of empirical broad-spectrum antibiotics and necessary combination therapy with antifungal drugs for children with respiratory infections, and timely adjustment of plans based on pathogen detection and drug sensitivity results. Regular respiratory care, such as nebulization inhalation, physical expectoration, positional drainage, necessary bronchoalveolar lavage, etc., is crucial for clearing respiratory secretions, improving ventilation, and reducing the burden of infection. Immunoglobulin infusion may help enhance the resistance of pediatric patients, especially in the presence of recurrent severe infections, but its long-term efficacy and indications still need further evaluation. Meanwhile, strictly controlling the ambient temperature, avoiding high heat, and reducing energy consumption are important measures to indirectly alleviate infection pressure.

At present, the treatment options for patients with XLHED are limited, which makes genetic counseling and prenatal diagnosis crucial for affected families. Prenatal diagnosis can be accurately diagnosed by detecting specific *EDA* variant through chorionic or amniocentesis (9). However, this procedure is invasive and carries relatively high risks. The cost of genetic testing is also relatively high, and it is not suitable for every individual being tested. Studies have shown that as early as the first 3 months of pregnancy, the fetal tooth germ can be detected by ultrasound, and in the middle and late stages of pregnancy, the range of tooth germ detected by ultrasound examination is larger. If at least one piece of alveolar bone has fewer than 6 tooth germs, it is considered that the number of tooth germs has decreased. Domestic and foreign literature reports that ultrasound examination of fetal tooth germ at 20–24 weeks of pregnancy can serve as valuable diagnostic clues (10, 11). For those with abnormal ultrasound examination, further amniocentesis for genetic testing of amniotic fluid is an economical and effective prenatal diagnostic approach.

With the popularization of genetic diagnosis, precise prenatal diagnosis of XLHED is no longer a technical challenge. However, there is currently no fundamental cure in terms of treatment, and some affected families choose to terminate their pregnancies due to their inability to accept severe phenotypes. It is encouraging that preclinical studies of the recombinant EDA A1 substitute protein (Fc EDA) have shown promising therapeutic potential. In the naturally occurring XLHED dog model, intravenous injection of Fc EDA after birth can not only correct developmental abnormalities in adult permanent teeth for a long time, but more importantly, it can repair congenitally missing tracheal and bronchial glands, improve mucociliary clearance function, and completely eliminate respiratory infections (12). The respiratory gland repair and infection disappearance effects of this dog model directly support the scientific hypothesis that prenatal protein replacement can reverse respiratory structural defects. Preliminary progress has also been made in current human clinical trials: a long-term follow-up study involving 9 male patients with obvious XLHED phenotype showed that patients who received Fc EDA treatment (*n* = 3) after birth did not detect sweat glands and sweating ability between 12 and 60 months of age. In contrast, patients who received prenatal treatment at 26 weeks of pregnancy and above (*n* = 6) showed good sweat gland development and pilocarpine-induced sweating, and had more permanent teeth than untreated relatives. The longest follow-up period was 6 years (13). In addition, Schneider et al. reported the results of prenatal treatment for three fetuses with XLHED in a “compassionate medication” study: two twins received one injection of Fc EDA into the amniotic cavity at 26 and 31 weeks of pregnancy, and normal sweat gland duct density and sweat volume were detected after delivery at 33 weeks. No XLHED-related fever or hospitalization occurred between 14 and 22 months of age. Another singleton received a single injection at 26 weeks of pregnancy and achieved normal sweating ability after delivery at 39 weeks (1). Their bone growth and tooth development returned to normal levels. No serious adverse reactions were observed in the above-mentioned human prenatal treatments, supporting the safety of Fc EDA. Based on current evidence, prenatal EDA1 replacement therapy is one of the most promising intervention strategies for XLHED and has entered further clinical trial validation stages (such as the Edelife study). Its potential to fully restore sweat gland function and reduce the risk of respiratory infections brings hope for fundamental treatment of the disease.

## 4 Conclusion

In summary, this study identified a novel frameshift variant c.916delC in the *EDA* through a detailed report of a child with XLHED and their family, further enriching the pathogenic variation spectrum of this gene. The presentation and family history of this case suggest that, in addition to abnormal development of skin appendages, incomplete development of respiratory mucosal glands and their resulting weak local defense function may be an important factor in recurrent and severe respiratory infections in infants and young children. Based on the diagnosis and treatment experience in this case, clinicians should be highly vigilant about the risk of severe pneumonia in such children. Early airway care, temperature regulation, and reasonable anti-infection support may help improve the prognosis for children. In addition, this study demonstrated the added value of combining genetic testing with prenatal screening for early diagnosis in high-risk families, suggesting that interdisciplinary collaborative management has potential clinical benefits for these patients.

## Data Availability

The datasets presented in this study can be found in online repositories. The names of the repository/repositories and accession number(s) can be found in the article/Supplementary Material.
