# Supplementary material for: Case Report: Fatal pneumonia as a manifestation of X-linked hypohidrotic ectodermal dysplasia: clinical phenotype and genotype characterization of a novel EDA frameshift variant in a four-generation pedigree
Source: Front Pediatr. 2026 Jun 3;14:1799743. doi: 10.3389/fped.2026.1799743 (PMC13272354; doi:10.3389/fped.2026.1799743)
Supplement: Supplementary file 1 [file Supplementaryfile1.pdf]

Attachment 1

Pathogenicity:

PM2_Supporting: This mutation has not been included in general population databases such as gnomAD, ChinaMAP, etc., indicating that it is extremely rare in the general population;

PVS1_Strong: This mutation is not expected to cause nonsense-mediated mRNA protein degradation and may result in encoding truncated proteins. Multiple other suspected pathogenic or pathogenic variants have been reported around this mutation, such as c.916C>A（p.Gln306His）、c.923A>G (p.Glu308Gly)、c.1119G>C(p.Met373Ile)、c.920T>G（p.Val307Gly）、c.916C>T(p.Gln306Ter)、c.922G>T（p.Glu308Ter）、c.920dup(p.Glu308Argfs*9) [PMID: 39408781, PMID: 33222196,18231121,29263514,40428344,38952411]， The protein region lost due to this mutation is crucial for protein function.

PP4: The phenotype of the subject is highly consistent;

PP1: The variant co-segregates with the XLHED phenotype across a four-generation pedigree. Five hemizygous males exhibit the classic triad and severe respiratory symptoms, while four heterozygous females show milder manifestations such as hypodontia and skin xerosis. The genetic findings perfectly align with the clinical phenotype in all nine tested members.

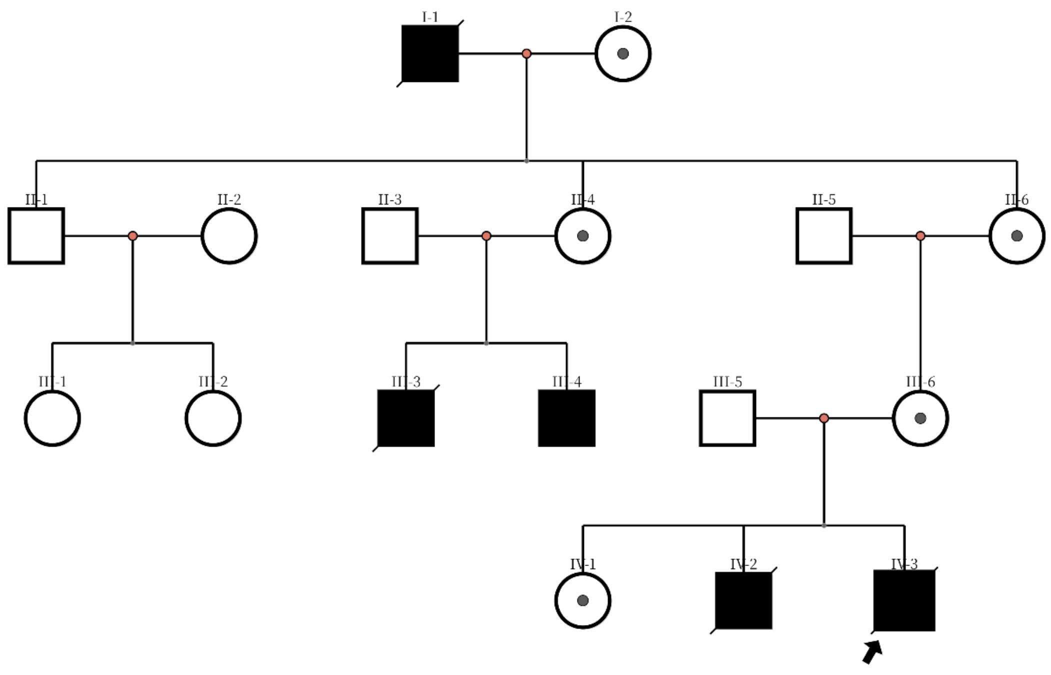

Based on PM2-Supporting+PVS1_Strong+PP4+PP1 as a suspected pathogenic variant
